# Comprehensive Analysis of LIN28A in Chinese Patients With Early Onset Parkinson’s Disease

**DOI:** 10.3389/fgene.2021.740096

**Published:** 2021-10-18

**Authors:** Xiaojing Gu, Yanbing Hou, Yongping Chen, Ruwei Ou, Bei Cao, Qianqian Wei, Lingyu Zhang, Wei Song, Bi Zhao, Ying Wu, Huifang Shang

**Affiliations:** Laboratory of Neurodegenerative Disorders, Rare Disease Center, Department of Neurology, West China Hospital, Sichuan University, Chengdu, China

**Keywords:** LIN28A, early onset Parkinson’s disease, rare variant, screening, burden analysis

## Abstract

A loss-of-function variant in Lin-28 Homolog A gene (*LIN28A* p. R192G, rs558060339) has been identified in two East Asian ancestry patients with early-onset PD (EOPD). Functional studies revealed that such a variant could lead to developmental defects and PD-related phenotype, and the phenotypes could be rescued after correction of the variant. The aim of the study was to screen the variants of *LIN28A* in Chinese patients with EOPD. A total of 682 EOPD patients were sequenced with whole exome sequencing and the coding and flanking region of *LIN28A* were analyzed. We identified a rare coding variant, p. P182L, of *LIN28A* in a Chinese patient with EOPD. Moreover, we also found a 3′-UTR polymorphism (rs4659441) to be associated with an increased risk for PD. However, our rare variant burden analysis did not support a role for *LIN28A* as a major causal gene for PD.

## Introduction

Parkinson’s disease (PD) is the second most common neurodegenerative disease with a complex spectrum of etiologies including aging, environmental factors, and genetic causes (Poewe et al., 2017). So far, more than 30 genes have been identified to be causative genes for PD, however, these could only explain a small proportion of patients (Shadrina et al., 1016).

With the development of genetic sequencing methods and bioinformatic analysis algorithms, novel PD causative genes have been extensively explored in recent years, and Lin-28 Homolog A gene (*LIN28A*) is one of them. LIN28A is a highly conserved RNA-binding protein, which is mainly expressed in the nervous system during early embryonic and fetal development and is involved in neuronal differentiation, tissue repair, and the maintenance of synaptic plasticity (Shinoda et al., 2013; Hu et al., 2020). Moreover, previous studies have also indicated that LIN28A might play a role in the pathogenesis of PD. For example, a previous study showed that LIN28A exhibits a strong therapeutic potential in the cell model and mouse model of PD (Rhee et al., 2016). A loss-of-function variant in the *LIN28A* (p.R192G, rs558060339) was identified in two East Asian ancestry patients with early-onset PD (EOPD) (Chang et al., 2019); developmental defects and PD-related phenotype due to the variant was rescued after correction of the variant (Chang et al., 2019). However, a subsequent study conducted in a large cohort of PD patients of European ancestry failed to find evidence supporting the causative role of *LIN28A* in PD (Diez-Fairen et al., 2021). There exists genetic heterogeneity among different regions. EOPD is more susceptible to genetic factors. Therefore, it is necessary to further investigate the role of *LIN28A* in EOPD.

## Materials and Methods

### Patients

A total of 682 EOPD patients (age of onset ≤45 years) admitted to the Department of Neurology, West China Hospital were recruited into the study. All the PD patients were diagnosed by experienced neurologists based on the established clinical diagnostic criteria for PD (Hughes et al., 1992; Postuma et al., 2015). Data of demographic, clinical characteristics, and rating scale assessments for patients were collected by face-to-face interview as previously described (Gu et al., 2020). Written informed consent was obtained from all participants. The study was approved by the ethics committee of West China Hospital, Sichuan University.

### Variant Selection

Genomic DNA was collected from peripheral blood leukocytes and underwent whole exome sequencing (WES). Procedures of WES and variants annotation were performed as previously described (Gu et al., 2020). All variants in the coding region and the flanking region of *LIN28A* captured by WES were included in the analysis. For the pathogenicity analysis, patients with mutations in known PD causative genes were excluded first.

### Single Nucleotide Polymorphism Association Analysis

Controls were from the gnomAD East Asian population (https://gnomad.broadinstitute.org/). The genotype and allele frequencies were compared between the patients and controls using a chi-square test, and a *p* value < 0.0056 (0.05/9) was considered as statistically significant after Bonferroni correction.

### Rare Variants Burden Analysis

Controls were from the gnomAD East Asian population (https://gnomad.broadinstitute.org/). All the rare coding variants annotated as “missense,” “splice donor,” “splice acceptor,” “splice region,” “stop-gained,” or “in-frame deletion” were included. Rare variants were defined by minor allele frequency (MAF) lower than 0.1% in the public database as mentioned above. Combined Annotation Dependent Depletion (CADD) integrates predictions from numerous bioinformatic algorithms into a single “C-score” and ranks all possible nucleotide changes in the genome based on potential to disrupt gene/protein function (Robak et al., 2017). In accordance with the previous study, we defined a stringent CADD C-score ≥ 12.37 as likely damaging variants, representing the top 2% most damaging of all possible nucleotide changes in the genome—this subset is enriched for known pathogenic alleles (Uitterlinden et al., 2017). All the rare and rare deleterious variants annotated as “missense,” “splice donor,” “splice acceptor,” “splice region,” “stop-gained,” or “in-frame deletion” were included. Five different algorithms method were used for burden analysis with AssotesteR Package in the rare variants level and rare deleterious variants level (Lee et al., 2014), including Comprehensive Approach to Analyzing Rare Genetic Variants (CARV), Sum of Squared Score (SSU), Sum Test (SUM), Cumulative Minor Allele Test (CMAT), and Bayesian Score Test (BST).

## Results

The coding region and the flanking region of *LIN28A* were captured by the WES. The likely pathogenic variant p. R192G was not detected in our cohort. In total, nine variants were detected in our cohort, including six intronic variants, a variant in the 3′-untranslated region (3′-UTR), a synonymous variant in exon 3 (c.270T > A, p. G90 =), and a missense variant in exon 4 (c.545C > T, p. P182L) (Table 1). The frequency of the missense variant is 0.0001 (2/17,248) in the gnomAD East Asian controls (PM2). The variant was predicted to be tolerated among several in silico prediction tools including SIFT (tolerated), PolyPhen2_HDIV (benign), PolyPhen2_HVAR (benign), and LRT (Neutral). Therefore, it was considered to be a variant of uncertain significance (VUS) (Richards et al., 2015). The patient carrying the p. P182L developed tremor and rigidity at the age of 16. He first visited our clinic at the age of 23 years old, and was comprehensively assessed. His Unified Parkinson’s Disease Rating Scale Part III score was 50, Horhn-Yahr stage was stage 3, and cognition was normal.

**TABLE 1:** The description of variants in *LIN28A* identified in the EOPD patients.

	gDNA	Amino acid change	dbSNP147	Frequency in patients	Frequency in gnomAD east Asia	Class	Status	p	Odds ratio (95% confidence interval)
1:26,738,110	C > G	—	rs202211941	0.00586 (8/1,364)	0.00013 (27/15378)	intronic	heterozygous	0.006	3.354 (1.521–7.398)
1:26,738,112	T > C	—	—	0.00073 (1/1,364)	—	intronic	heterozygous	—	—
1:26,738,117	G > A	—	—	0.00073 (1/1,364)	—	intronic	heterozygous	—	—
1:26,738,122	C > T		rs4623750	0.06871 (94/1,364)	0.08406 (1,250/14870)	intronic	heterozygous	0.052	0.806 (0.649–1.002)
1:26,738,123	delA	—	—	0.00073 (1/1,364)	—	intronic	heterozygous	—	—
1:26,751,835	T > A	p.G90 =	-	0.00073 (1/1,364)	-	synonymous	heterozygous	-	-
1:26,751993	G > A	—	rs187064721	0.00147 (2/1,364)	0.00413 (71/17184)	intronic	heterozygous	0.052	0.177 (0.025–1.274)
1:26,752,864	C > T	p.P182L	rs769630938	0.00073 (1/1,364)	0.00011 (2/17248)	missense	heterozygous	0.204	6.362 (0.573–69.814)
1:26,752,992	T > C	—	rs4659441	0.15322 (209/1,364)	0.11879 (1947/16390)	3′-UTR	heterozygous	<0.001	1.132 (1.150–1.567)

Adjusted *p* = 0.05/9 = 0.0056.

In addition to the coding variants, there were seven non-coding variants identified in the flaking region. At the allelic level, rs4659441 in the 3′-UTR was found to be associated with an increased risk for EOPD (Table 1).

However, in the gene-based rare variant burden analysis, there was no significant enrichment of rare coding variants/rare damaging coding variants in Chinese patients with EOPD when compared with the gnomAD East Asian controls (Supplementary Table S1 and Supplementary Table S2).

## Discussion

In the current study, we identified a rare coding variant p. P182L in the *LIN28A* in a Chinese patient with juvenile onset PD. Moreover, we found a polymorphism in the 3′-UTR to be associated with an increased risk for PD. However, rare coding variant burden analysis did not support *LIN28A* as a major causative gene for PD.

The rare coding variant p. P182L identified in our study, together with the previously described loss-of-function variant p. R192G found in two East Asian ancestry patients with EOPD (Chang et al., 2019), and the rare coding variant p. T189I identified in a European PD patient (Diez-Fairen et al., 2021), were all located in the exon 4 of *LIN28A*, which is the C-terminal domain, distal to an RNA-binding Zn-knuckle domain (residues 138–176) (Chang et al., 2019). Therefore, exon 4 might be a mutated hot spot for *LIN28A*, although more functional studies are needed. Our patient carrying p. P182L had a much earlier age of onset (at the age of 16) than that of the Korean patient carrying p. R192G (at the age of 23 and 60).

In addition to the rare coding variants identified in the current study, rs4659441 in the 3′-UTR was found to be associated with an increased risk for EOPD. Although untranslated, the 3′-UTR is important in the regulation of mRNA-based processes including mRNA localization, mRNA stability, and translation (Mayr, 2019). More specifically, 3′UTR can provide binding sites to certain miRNAs and lead to mRNA degradation, therefore inhibiting gene expression (Mayr, 2019). *Via* bioinformatic tools (http://www.mirbase.org/search-rnacentral.shtml) (Kozomara et al., 2019), we found that rs4659441 was a binding site for both hsa-miR-4476 and hsa-miR-505-5p. Interestingly, hsa-miR-505-5p, also known as hsa-miR-505*, has been found to be alter-regulated in a wide spectrum of neurological disorders including multiple sclerosis, myasthenic gravis, Alzheimer’s disease, Fredrich Ataxia, and Lacunar stroke (http://bio-bigdata.hrbmu.edu.cn/nsdna/map. jsp?organism = Homo%20sapiens). In relation to PD, hsa-miR-505-5p has been found to be upregulated in the cell model of PD (Li et al., 2013), and in the brain of progressive supranuclear palsy, a parkinsonism plus syndrome (Ubhi et al., 2014). Therefore, it can be speculated that the hsa-miR-505-5p could regulate the expression of *LIN28A via* binding to the polymorphism of rs4659441 in the 3′-UTR, and thus contribute to the pathogenesis of PD. However, further functional studies are needed to elucidate the role of rs4659441.

Last but not least, burden analysis is a method that calculates the aggregated effect of a gene on some diseases. However, our findings using mathematical methods did not indicate the enrichment of rare variants in *LIN28A* in EOPD patients, which is consistent with the negative findings from the European population (Diez-Fairen et al., 2021).

## Conclusion

In conclusion, our findings of a rare coding variant p. P182L expanded the mutation spectrum of *LIN28A* in EOPD. Moreover, we also found a risk allele for EOPD in the 3′-UTR. However, our rare variant burden analysis together with the findings from the European population did not support the hypothesis that *LIN28A* plays a major causative role in PD. More studies in different genetic backgrounds are needed.

## Data Availability

The original contributions presented in the study are included in the article/Supplementary Material, further inquiries can be directed to the corresponding author.
