# Supplementary material for: Comprehensive Analysis of LIN28A in Chinese Patients With Early Onset Parkinson’s Disease
Source: Front Genet. 2021 Oct 18;12:740096. doi: 10.3389/fgene.2021.740096 (PMC8558378; doi:10.3389/fgene.2021.740096)
Supplement: Supplementary file 1 [file DataSheet1.DOCX]

***Supplementary Table 1*** Rare variants and rare damaging rare variants in patient and control for burden analysis.

| Chromosome | Position | rsID | Reference | Alternate | CADD | Allele Count East Asian | Total Allele in East Asian | frequency |
| --- | --- | --- | --- | --- | --- | --- | --- | --- |
| Control in gnomAD East Asian population | | |  |  |  |  |  |  |
| 1 | 26737892 | rs199880616 | C | T | 22.5 | 4 | 16086 | 0.00024866 |
| 1 | 26737895 | rs1484676472 | C | T | 22.9 | 1 | 16150 | 6.192E-05 |
| 1 | 26737900 | rs754594227 | G | A | 19.73 | 1 | 16206 | 6.1706E-05 |
| 1 | 26737927 | rs772081318 | G | C | 18.35 | 1 | 17044 | 5.8672E-05 |
| 1 | 26737930 | rs1345478758 | G | C | 18.8 | 1 | 17228 | 5.8045E-05 |
| 1 | 26738038 | rs1457334602 | G | T | 22.7 | 1 | 18278 | 5.4711E-05 |
| 1 | 26751805 | rs1447402085 | C | A | 22.4 | 1 | 18386 | 5.4389E-05 |
| 1 | 26751884 | rs769951372 | A | C | 16.91 | 2 | 18394 | 0.00010873 |
| 1 | 26751887 | rs749445188 | C | A | 23.7 | 3 | 18392 | 0.00016311 |
| 1 | 26751888 | rs771973442 | G | A | 26 | 4 | 18390 | 0.00021751 |
| 1 | 26751942 | rs141345172 | G | A | 25.9 | 1 | 18376 | 5.4419E-05 |
| 1 | 26751959 | rs143830906 | C | G | 25.6 | 2 | 18372 | 0.00010886 |
| 1 | 26752779 | rs1181611791 | C | G | 24.3 | 1 | 18394 | 5.4366E-05 |
| 1 | 26752864 | rs769630938 | C | T | 23.5 | 2 | 18394 | 0.00010873 |
| 1 | 26752888 | rs768055786 | A | G | 21.5 | 1 | 18394 | 5.4366E-05 |
| 1 | 26752893 | rs558060339 | C | G | 22.9 | 8 | 18394 | 0.00043492 |
| 1 | 26752896 | rs764543175 | G | C | 22.2 | 1 | 18394 | 5.4366E-05 |
| Patient |  |  |  |  |  |  |  |  |
| 1 | 26752864 | rs769630938 | C | T | 23.5 | 1 | 1364 | 0.00073314 |

***Supplementary Table 2*** Burden analysis results for rare/rare damaging variants in *LIN28A.*

| Algorithm | p value for rare variants burden analysis | p value for rare damaging variants burden analysis |
| --- | --- | --- |
| CARV | 0.098 | 0.098 |
| SSU | 0.258 | 0.258 |
| SUM | 0.698 | 0.698 |
| CMAT | 0.258 | 0.258 |
| BST | 0.258 | 0.258 |

CARV: Comprehensive Approach to Analyzing Rare Genetic Variants; SSU: Sum of Squared Score; SUM: Sum Test (SUM); CMAT: Cumulative Minor Allele Test; BST: Bayesian Score Test.
